# Fast real-time detection and counting of thrips in greenhouses with multi-level feature attention and fusion

**DOI:** 10.3389/fpls.2025.1663813

**Published:** 2025-08-21

**Authors:** Zhangzhang He, Xinyue Chen, Ying Gao, Yu Zhang, Yuheng Guo, Tong Zhai, Xiaochen Wei, Huan Li, Haipeng Zhu, Yongkun Fu, Zhiliang Zhang

**Affiliations:** ^1^ College of Food and Biology, Jingchu University of Technology, Jingmen, Hubei, China; ^2^ School of Computer Science, Yangtze University, Jingzhou, China; ^3^ Ministry of Agriculture and Rural Affairs of the People’s Republic of China (MARA) Key Laboratory of Sustainable Crop Production in the Middle Reaches of the Yangtze River (Co-Construction by Ministry and Province), College of Agriculture, Yangtze University, Jingzhou, China

**Keywords:** thrip, pest counting, pest detection, precision agriculture, lightweight network

## Abstract

Thrips can damage over 200 species across 62 plant families, causing significant economic losses worldwide. Their tiny size, rapid reproduction, and wide host range make them prone to outbreaks, necessitating precise and efficient population monitoring methods. Existing intelligent counting methods lack effective solutions for tiny pests like thrips. In this work, we propose the Thrip Counting and Detection Network (TCD-Net). TCD-Net is an fully convolutional network consisting of a backbone network, a feature pyramid, and an output head. First, we propose a lightweight backbone network, PartialNeXt, which optimizes convolution layers through Partial Convolution (PConv), ensuring both network performance and reduced complexity. Next, we design a lightweight channel-spatial hybrid attention mechanism to further refine multi-scale features, enhancing the model’s ability to extract global and local features with minimal computational cost. Finally, we introduce the Adaptive Feature Mixer Feature Pyramid Network (AFM-FPN), where the Adaptive Feature Mixer (AFM) replaces the traditional element-wise addition at the P level, enhancing the model’s ability to select and retain thrips features, improving detection performance for extremely small objects. The model is trained with the Object Counting Loss (OC Loss) specifically designed for the detection of tiny pests, allowing the network to predict a small spot region for each thrips, enabling real-time and precise counting and detection. We collected a dataset containing over 47K thrips annotations to evaluate the model’s performance. The results show that TCD-Net achieves an F1 score of 85.67%, with a counting result correlation of 75.50%. The model size is only 21.13M, with a computational cost of 114.36 GFLOPs. Compared to existing methods, TCD-Net achieves higher thrips counting and detection accuracy with lower computational complexity. The dataset is publicly available at github.com/ZZL0897/thrip_leaf_dataset.

## 1 Introduction

Thrips belong to the order Thysanoptera and the family Thripidae. These insects are small in size, reproduce rapidly, and have a body length of less than 2mm. They are typically yellow, brown, or black in color. The eggs vary in shape, including kidney-shaped, round, and oval, with colors ranging from colorless to white and yellow (Zhang, 2011; Wu et al., 2018). Thrips exhibit diverse feeding habits, predominantly phytophagous. Thrips exhibit diverse feeding habits, predominantly phytophagous. They can damage over 200 crop species from 62 families, including Cucurbitaceae, Fabaceae, Brassicaceae, and Solanaceae (Kirk et al., 2021). Thrips inflict significant economic losses worldwide. Controlling thrips is challenging for three main reasons: 1) Their small size and strong concealment tendencies, as they prefer to hide in flowers, tender tips, and the undersides of leaves, making detection difficult. 2) Their short life cycle and rapid reproduction, which contribute to the rapid development of resistance to chemical pesticides, leading to outbreaks. 3) Their broad host range, strong dispersal ability, and excellent ecological adaptability, enabling severe damage to various crops (Steenbergen et al., 2018). Therefore, It is crucial to accurately detect and count thrips.

Traditional manual counting methods for pests are time-consuming and labor-intensive, while computer vision and deep learning-based intelligent detection technologies can significantly improve monitoring efficiency (Zhang et al., 2020b, 2024, 2024; Liu et al., 2025; Zhang et al., 2025). Current research on pest intelligent detection and counting mainly focuses on improvements to object detection algorithms. Key improvements include optimizing feature extraction backbones, enhancing the Feature Pyramid Network (FPN), improving the Region Proposal Network (RPN), and optimizing anchor generation and selection mechanisms to better suit pest counting and detection tasks (Jiao et al., 2020; Dong et al., 2021; Liu et al., 2021a; Wang et al., 2021b; Jiao et al., 2022b; Wang et al., 2023; Dong et al., 2024b). For instance, Wang et al. (2021a) and Jiao et al. (2022a) both made improvements to R-CNN by incorporating attention mechanisms into the network, enriching the features extracted to enhance detection performance. Dong et al. (2024a) made comprehensive improvements to the YOLO model, effectively enhancing the model’s feature attention capabilities and multi-scale feature extraction, increasing accuracy while reducing model parameters. These studies demonstrated the strong benchmark performance of object detection in pest counting and detection tasks. They have made effective improvements to address challenges such as small pest size and complex backgrounds, promoting the application of object detection methods in agricultural pest detection expert systems.

However, detecting and counting extremely small pests like thrips and planthoppers still poses challenges. Small object detection has consistently posed a challenge for object detectors, often resulting in False Negatives (FNs) and False Positives (FPs). The limited features and low signal-to-noise ratio of extremely small pests hinder object detectors from extracting sufficient features or accurately locating the anchors (Zhan et al., 2022; Dong et al., 2024b; Zhang et al., 2024a). Some scholars have explored solutions to these challenges. He et al. (2020) and Lee et al. (2020) both used Faster R-CNN for intelligent detection of brown planthoppers and tea thrips, respectively. Wang et al. (2021a) and Wang et al. (2021b) improved RPN and incorporated feature attention mechanisms to enhance detection performance for small pests. De Cesaro et al. (2022) utilized Mask R-CNN for counting aphids and parasitic wasps, achieving approximately 80% result correlation. Li et al. (2022) proposed a two-stage detection method for whiteflies and thrips, initially locating pests using spectral features, followed by recognition using Support Vector Machines (SVM). Wang et al. (2023) developed an anchor-free framework and a dynamic detection head, achieving competitive results on two multi-class small-object pest datasets. Dong et al. (2024b) designed multi-scale feature aggregation and dynamic perception modules, achieving optimal detection performance. Yang et al. (2024) introduced a super-resolution module and multi-level feature fusion in YOLOv8, achieving a 57% mAP for detecting extremely small pests. Zhang et al. (2024a) proposed an innovative rice planthopper detection method based on a fully convolutional architecture and object counting loss, achieving an F1 score of 92.36%. Banerjee et al. (2024) and Wu et al. (2024) designed IoT-based thrips pest monitoring systems, which effectively improved monitoring efficiency for thrips populations in their experimental environments.

The aforementioned studies provide innovative research ideas and improvement pathways for counting and detecting extremely small pests. However, research on intelligent counting methods for thrips remains limited. Existing methods for precise counting and detection of thrips still have significant room for improvement in detection accuracy and model runtime efficiency. Therefore, this paper focuses on thrips as the research subject, collects thrips infestation data from *Spathiphyllum floribundum ‘Clevelandii’* cultivated in greenhouses, and proposes a new real-time counting and detection algorithm for thrips, offering an efficient and reliable intelligent method for monitoring small pests in greenhouses. The main contributions of this paper are as follows:

1. Thrip Counting and Detection Network (TCD-Net). A fully convolutional network based on a multi-level attention mechanism and feature adaptive fusion is built. The Object Counting Loss (OC Loss), designed for extremely small pests, is used to train the network, enabling real-time and accurate detection and counting of thrips in greenhouses.
2. Optimized backbone network and feature attention mechanism. The PartialNeXt backbone network is proposed, the convolution layers of ConvNeXtV2 are optimized using Partial Convolution (PConv), improving the network’s computational efficiency and feature reuse capability. Then, a channel-spatial hybrid attention (HA) mechanism that balances performance and efficiency is designed to enhance detection stability.
3. Multi-scale feature adaptive fusion: The Adaptive Feature Mixer Feature Pyramid Network (AFM-FPN) is proposed, using Adaptive Feature Mixer (AFM) for adaptive fusion of P-level multi-scale features, enhancing the model’s ability to select and retain thrips features, thereby improving detection accuracy for extremely small objects.
4. We collect a thrips dataset consisting of 5,618 images and 47,726 annotations. Extensive experiments and comparisons are conducted on this dataset to verify the superiority of TCD-Net in detection accuracy and computational efficiency.

## 2 Materials

### 2.1 Data acquisition

**Figure 1:**
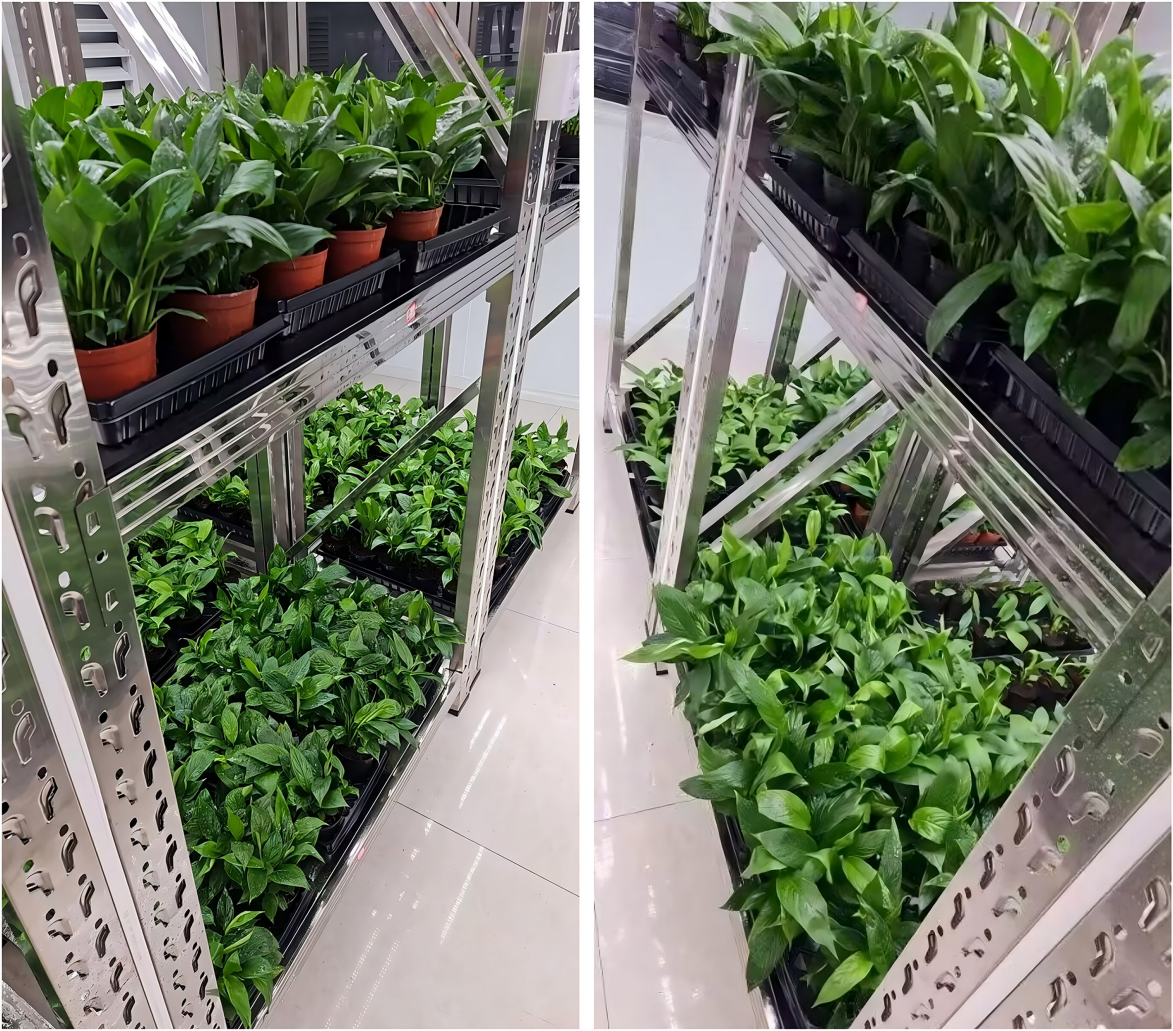
Plant greenhouse.

Our team collected the dataset from July to September 2024 in the Plant Growth Chamber at Jingchu Sci-tech Park, Jingchu University of Technology, using potted *Spathiphyllum floribundum ‘Clevelandii’*. The temperature in the growth chamber was 25°C, with humidity levels ranging from 50% to 70%, and light intensity was 10,000 lux. The thrips species identified on the infected leaves was *Megalurothrips usitatus*. Data collection was carried out by six plant protection students. They randomly took 2–3 images of thrips on the leaves at different time intervals using smartphones, keeping only the clearest image at each location. The shooting environment is shown in **Figure 1**.

### 2.2 Dataset

After data collection was completed, a total of 5,618 images were selected to form the dataset, and all images were resized to a resolution of 1280×1280. The thrips annotations were performed collaboratively by six photographers, followed by a second round of verification to ensure annotation accuracy. The annotation tool used was Labelme, with the initial annotation results in json format. Subsequently, we converted the annotation results to COCO and YOLO formats for easy comparison with other methods. The dataset contains a total of 47,726 thrips annotations. The dataset was split into training, validation, and test sets in a 6:2:2 ratio, and specific statistics are shown in **Table 1**. The dataset is publicly available at github.com/ZZL0897/thrip_leaf_dataset.

**Table 1:** Dataset information.

Train		Validation		Test		Statistics	
Images	Annotations	Images	Annotations	Images	Annotations	Avg. num	Avg. bbox area
3370	28934	1124	9407	1124	9385	8.5	176px

It is worth noting that the average pixel area of the thrips bounding boxes in the images is only 176px, with widths ranging from 2px to 54px and heights ranging from 2px to 56px. The ratio of the average pixel area of the bounding boxes to the image pixel area is only 0.011%, which highlights the fact that thrips are extremely small targets in the images, making accurate detection a significant challenge.

## 3 Proposed method

### 3.1 Network construction

**Figure 2:**
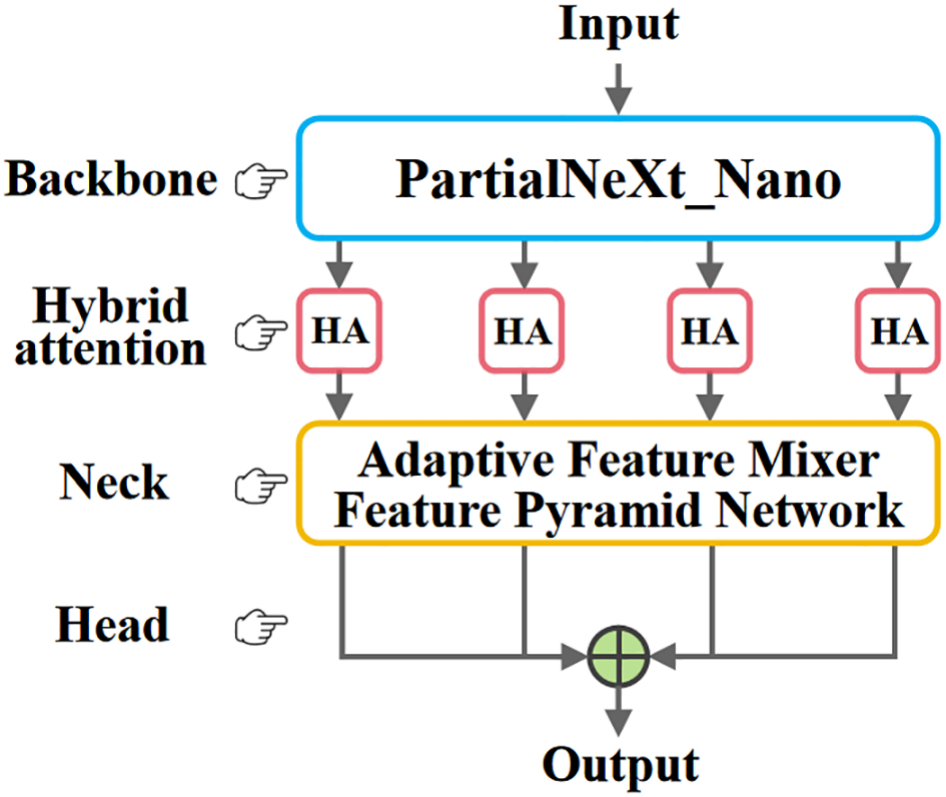
The overall structure of TCD-Net. The network architecture consists of four components. The backbone network extracts fundamental image features and outputs four sets of multi-scale feature maps. These four feature maps are then fed into the Hybrid Attention (HA) for further refinement, followed by adaptive feature fusion through the AFM-FPN. Finally, four 1×1 convolutional layers serve as the output heads to generate the prediction results.

The overall structure of TCD-Net is shown in **Figure 2**. Its modular design is similar to that of a typical object detection network. The backbone network extracts rich multi-scale feature information from the input image, with attention mechanisms further enhancing the feature representation. These multi-scale features are fed into the FPN to improve the network’s performance in detecting small objects (Lin et al., 2017). Finally, the output head generates the final predictions. Unlike traditional object detection methods, this network is fully convolutional. The output head consists of four 1×1 convolutions, which reduce the output channel count of the FPN to 1, and interpolate it back to the input size, ultimately combining the results into a single prediction output.

A regular fully convolutional network cannot count and localize tiny objects. We address this by using a specially designed loss function during training, allowing the network to accept object detection labels and enabling the counting and detection of small pests in images. The implementation process will be detailed in Sections 3.2 and 3.3.

#### 3.1.1 Feature extraction backbone

**Figure 3:**
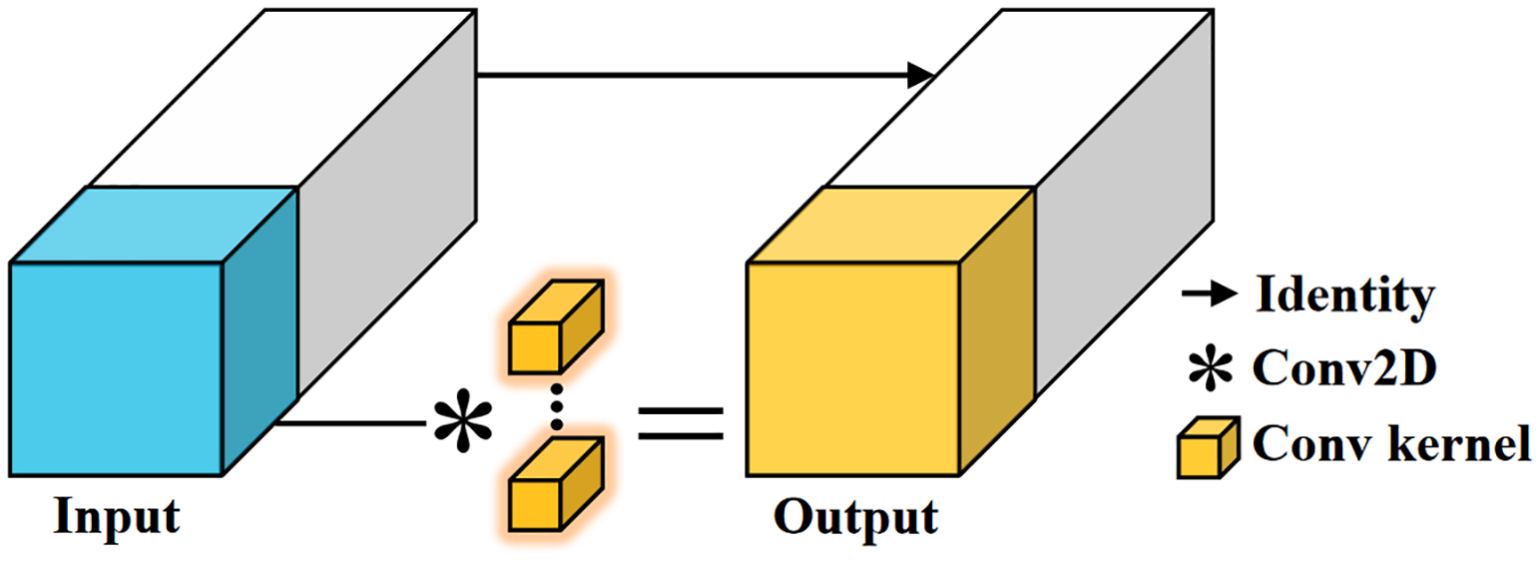
Partial convolution. PConv only performs traditional convolution operations on a small portion of the feature map, and the rest is directly passed to the next layer. This reduces computational redundancy and memory access frequency, and with the use of traditional convolutions, it benefits from better hardware support, improving computation speed.

The choice of feature extraction backbone plays a crucial role in the performance of the model. We improve the ConvNeXtV2 and propose the PartialNeXt, which offers higher computational efficiency and better feature extraction performance. The introduction of ConvNeXtV2 has elevated the convolutional neural network model to new heights in both computational efficiency and model performance (Woo et al., 2023). However, its key feature extraction convolution layer uses a 7×7 Depthwise Convolution (DWConv), which reduces the model’s parameter count and computation load. But due to increased memory access frequency and insufficient hardware optimization, the computational speed is actually reduced. Therefore, we replace the DWConv in ConvNeXtV2 with Partial Convolution (PConv) to enhance the model’s computational speed. The core idea of PConv is that there is significant redundancy in the massive feature maps of the model. PConv performs traditional convolution operations only on a small portion of the feature map, while the remaining majority of the feature map is directly passed to the next layer. This achieves a balance between model efficiency and performance (Chen et al., 2023). The operation process of PConv is shown in **Figure 3**.

**Figure 4:**
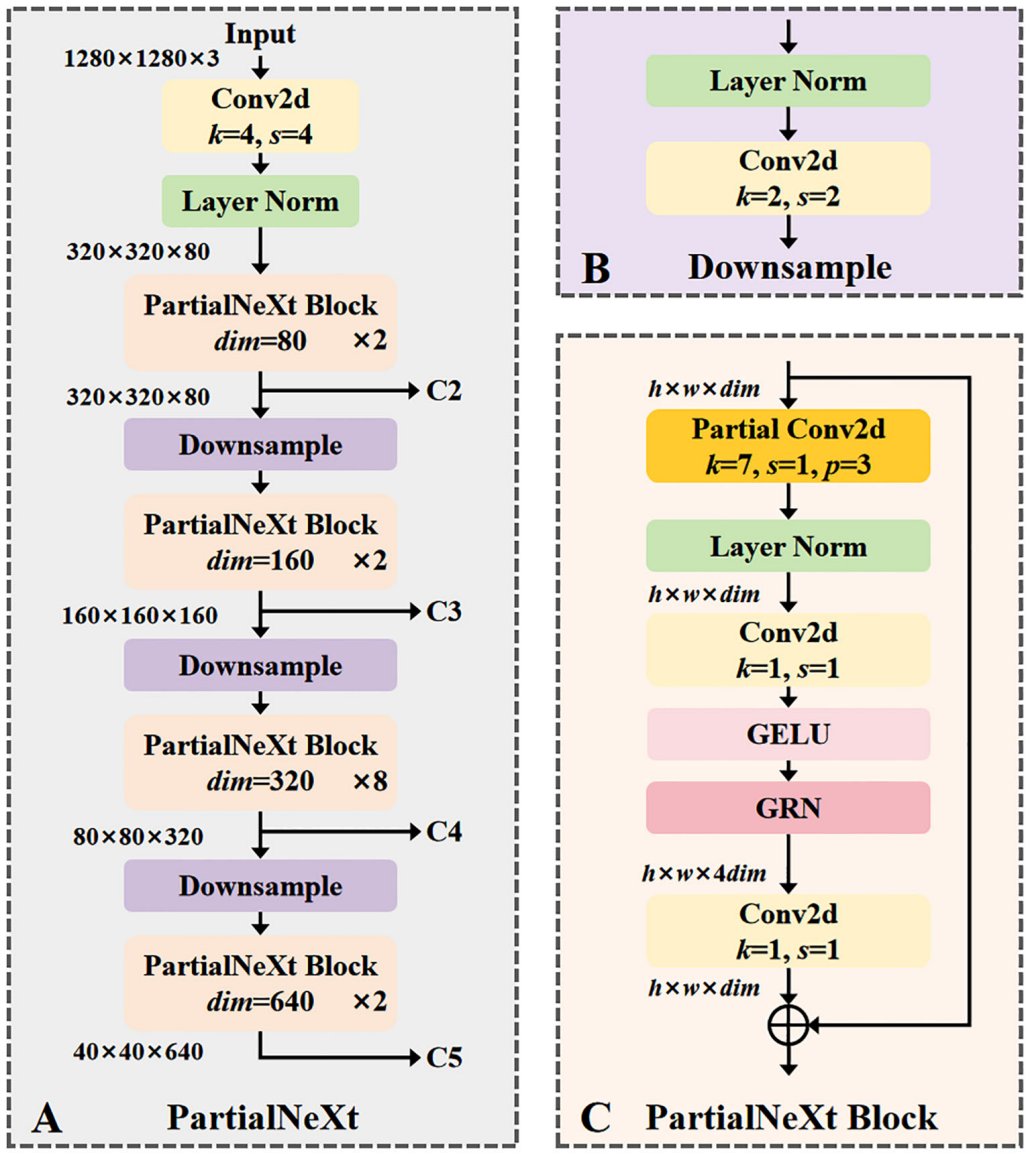
**(A)** The overall structure of PartialNeXt, its layers and channels are designed according to ConvNeXt Nano; **(B)** The structure of the Downsample layer; **(C)** The structure of the PartialNeXt Block, its key improvement is to use partial convolution to optimize feature extraction.

The structural parameters of the backbone network refer to the Nano version of ConvNeXtV2, which offers good feature extraction ability while maintaining low parameter and computation counts. The overall structure of PartialNeXt is shown in **Figure 4A**.

The network structure of PartialNeXt adopts a hierarchical design, divided into four stages. Each stage contains a downsampling layer, with the number of blocks and channels in each stage consistent with ConvNeXtV2 Nano. The stages, from shallow to deep, contain [2, 2, 8, 2] PartialNeXt Blocks with corresponding channel counts of [80, 160, 320, 640]. Multi-scale features are crucial for object detection tasks, and these four stages can extract features at four different scales, C2 to C5, for subsequent feature fusion. The structure of the Downsample layer is shown in **Figure 4B**, responsible for reducing the resolution of feature maps and expanding the channel count. At the beginning of each stage, a convolution layer with a kernel size of 2 and a stride of 2 reduces the resolution of the feature map by half while doubling the number of channels. Layer Normalization is applied to ensure stable feature distribution, enhancing model training efficiency. The structure of the PartialNeXt Block is shown in **Figure 4C**. Each Block starts with PConv, which is the most critical improvement, with a kernel size of 7. We use the default parameters from the PConv paper, where the ratio of the feature map for feature extraction to the feature map for direct forward is 1:3. A 1×1 convolution is used for cross-channel information fusion, while the other modules follow the ConvNeXtV2 design.

#### 3.1.2 Hybrid attention

Although the model employs a fully convolutional architecture, its objective is to achieve accurate counting and localization of tiny thrips rather than pursuing precise contour segmentation. Therefore, we introduce a lightweight hybrid channel-spatial attention mechanism. This mechanism focuses on enhancing the detection accuracy for small targets while introducing only minimal additional computational overhead. After the backbone network outputs four multi-scale features (C2–C5), all are fed into the HA module for feature extraction.

For the input feature *f*, we first compute its channel attention, then calculate its spatial attention, and finally add its residual, as shown in Equation 1. Below, we will detail the channel attention and spatial attention mechanisms.


(1) HA(f)=SA(CA(f))+f

**Figure 5:**
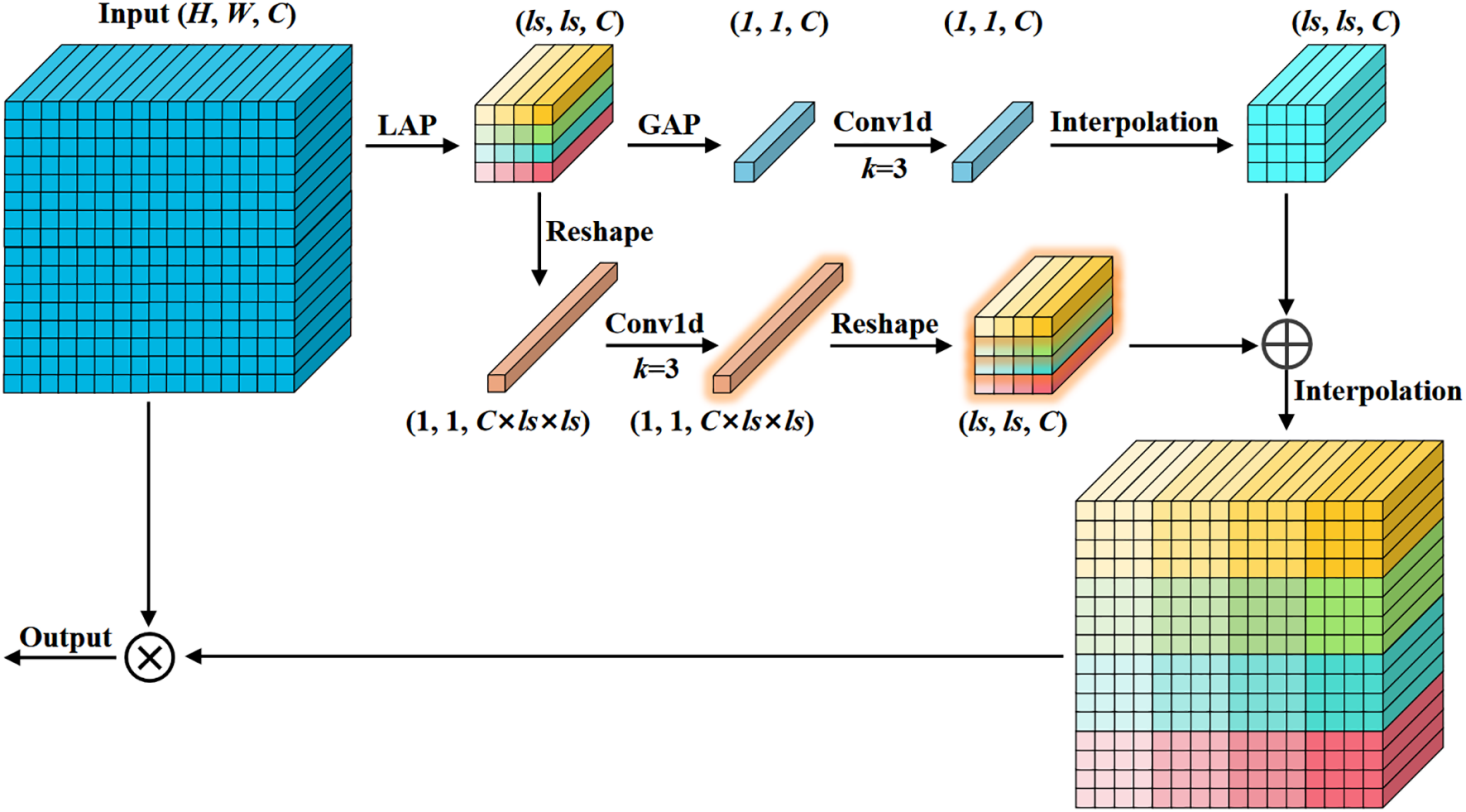
Mixed local channel attention. Integrating local and global features by using average pooling of different sizes in channel attention.

Most channel attention mechanisms apply global average pooling to the feature map, which captures only single-channel information. This offers limited improvements for detecting small objects, as global pooling tends to weaken the features of tiny targets. In our channel attention mechanism, we combine both local and global features to enhance performance on small objects while keeping computational overhead minimal (Wan et al., 2023). As shown in **Figure 5**, the input feature map undergoes adaptive average pooling to produce a local pooling result of size ls, followed by global average pooling applied to the local result to obtain the global pooling result. Local pooling emphasizes local region features, while global pooling captures the distribution characteristics of the entire feature map. Both local and global pooling results are passed through a 1D convolution to extract features and compute attention. The global attention is interpolated to the size of the local attention and fused by element-wise addition. Finally, the fused result is interpolated to the input size and multiplied with the input feature map to generate the final channel attention map.

**Figure 6:**
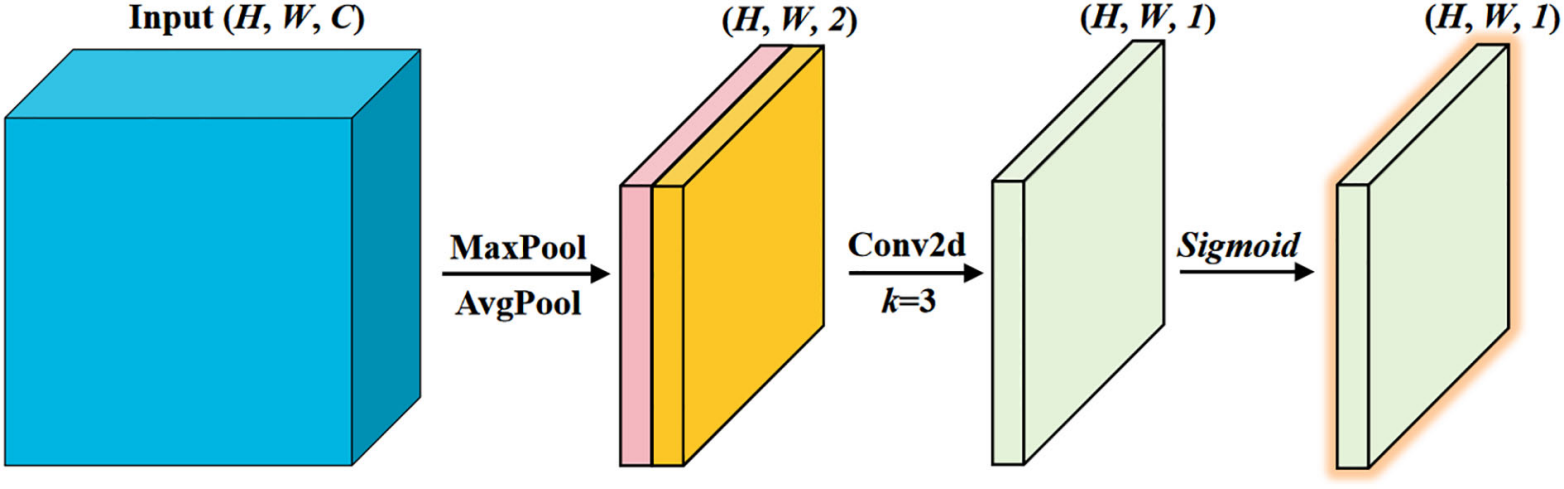
Spatial attention.

The implementation of spatial attention is straightforward. We adopt the spatial attention module from the Convolutional Block Attention Module (CBAM) (Woo et al., 2018), which incurs minimal computational overhead, as shown in **Figure 6**. First, we extract distribution information of the spatial features by performing average and max pooling along the channel dimension. Then, a 2D convolution is applied to compute spatial attention.

#### 3.1.3 Adaptive feature mixer feature pyramid network

**Figure 7:**
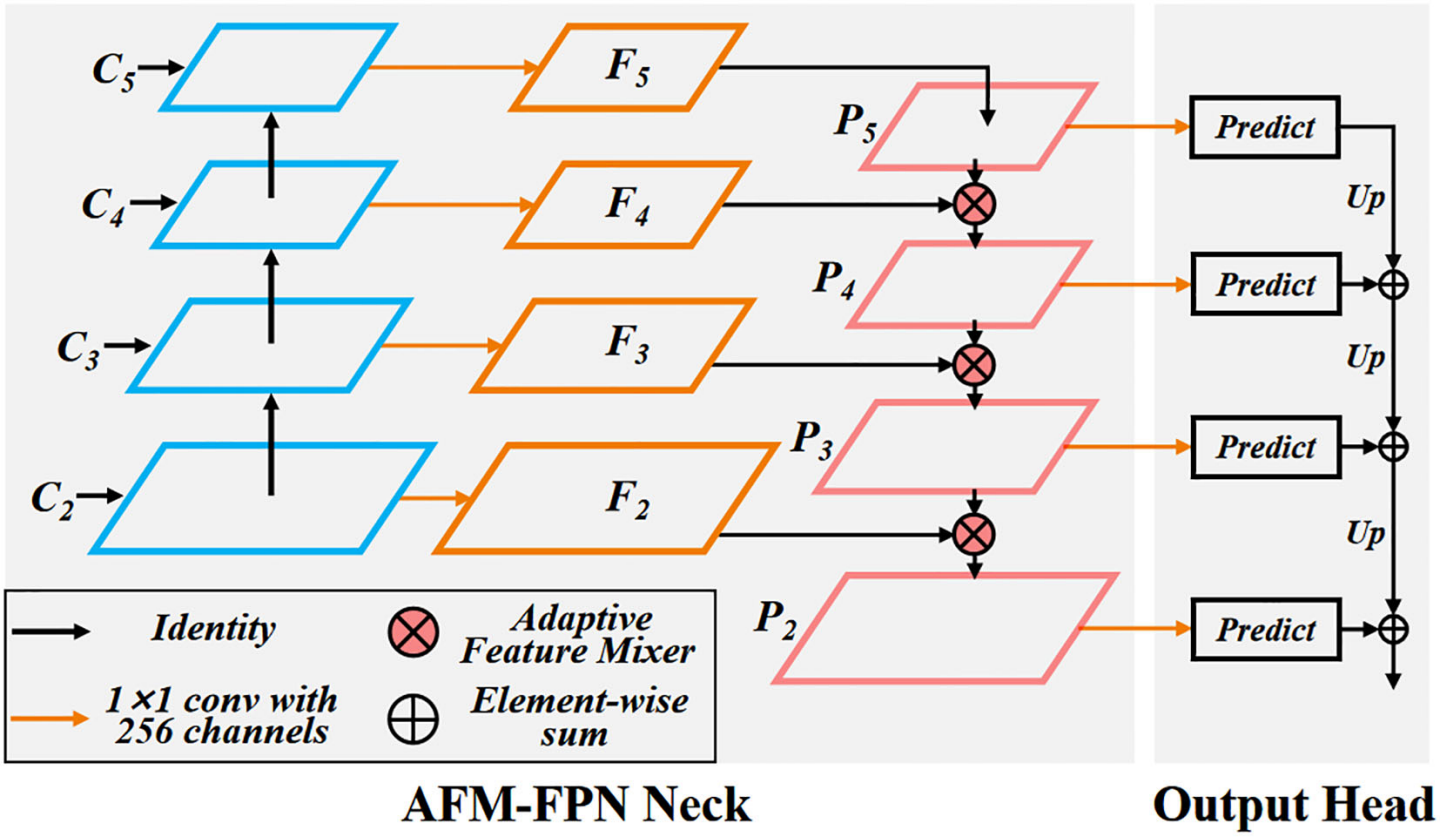
Adaptive feature mixer feature pyramid network. Optimizing the traditional element wise addition method for P-level features to use AFM module for feature adaptive fusion to enhance performance.

Feature Pyramid Networks (FPN) have become a standard paradigm for small object detection tasks, as they enhance small object feature information (Lin et al., 2017). Traditional FPNs fuse features through sampling and element-wise addition. However, this fusion method is not conducive to the flow of information between multi-scale feature maps. The element-wise addition could lead to the accumulation of abnormal feature information or cause the weakening of important features (Dai et al., 2021). To address this issue, we propose the Adaptive Feature Mixer Feature Pyramid Network (AFM-FPN). AFM-FPN uses an Adaptive Feature Mixer (AFM) module to perform adaptive weighted fusion of features, as shown in **Figure 7**.

**Figure 8:**
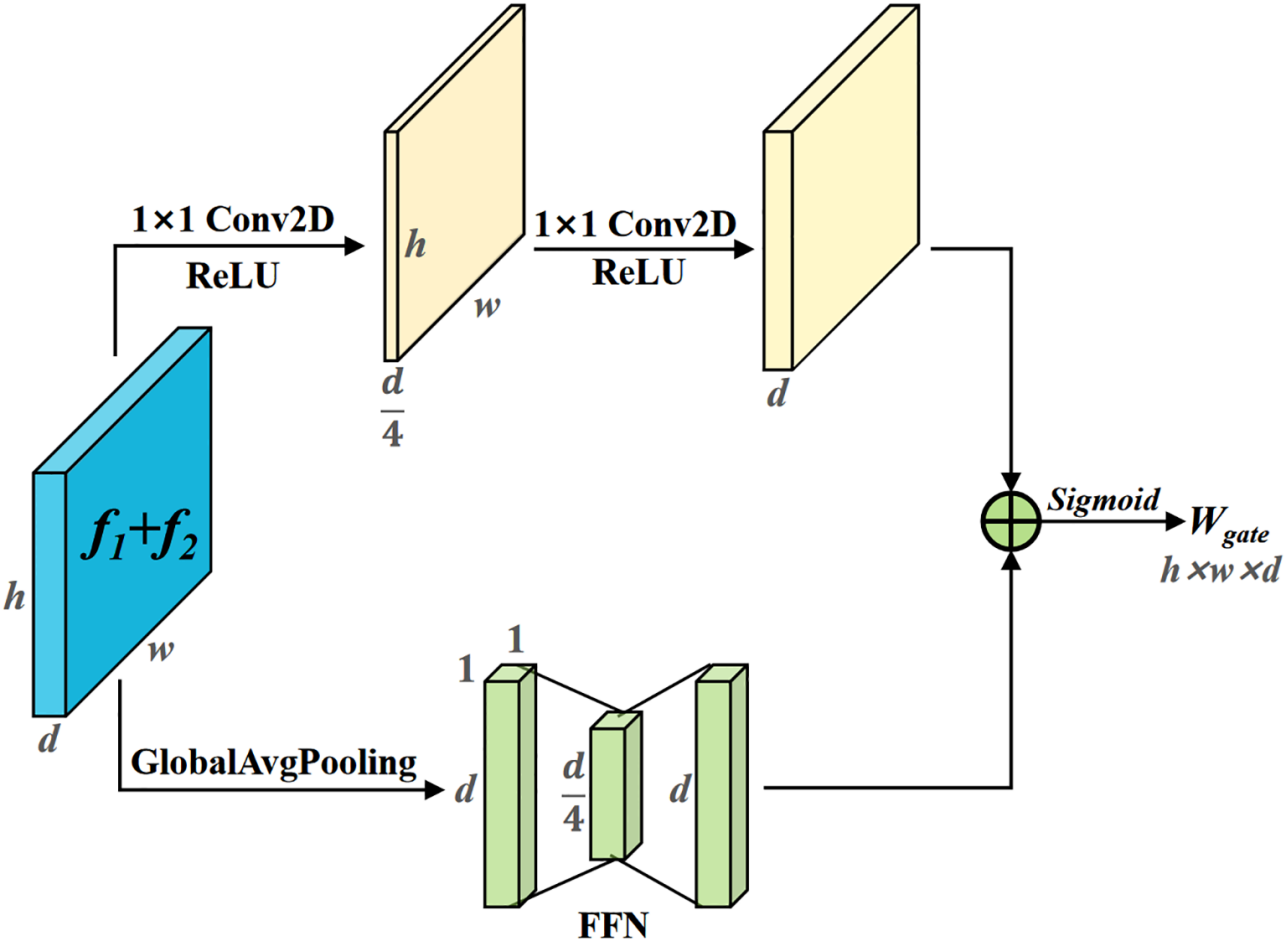
Adaptive feature mixer. By extracting the spatial and channel features of the input features, fusion weights are assigned pixel-wise for the two features to be fused.


The AFM module is divided into two branches: spatial feature extraction and channel feature extraction. It assigns fusion weights on a pixel-by-pixel basis for the two features to be fused, as shown in **Figure 8**. The two features to be fused are then added element-wise. Two 1×1 convolutions are used to obtain spatial feature weights with size (h, w, d). Global average pooling is applied to compress the spatial size of the feature map to 1×1, and a Feed Forward Network (FFN) is used to encode the channel feature weights. The channel feature weights are broadcasted and added element-wise with the spatial feature weights, followed by activation with the *Sigmoid* function to obtain the adaptive fusion weight *W*, with size (h, w, d). The features *f_1_* and *f_2_* are then weighted and fused using *W*, as shown in Equation 2.

(2) $$AFM\left(f_{1},f_{2}\right)=f_{1}\times W+f_{2}\times \left(1-W\right)$$

### 3.2 Loss function

**Figure 9:**
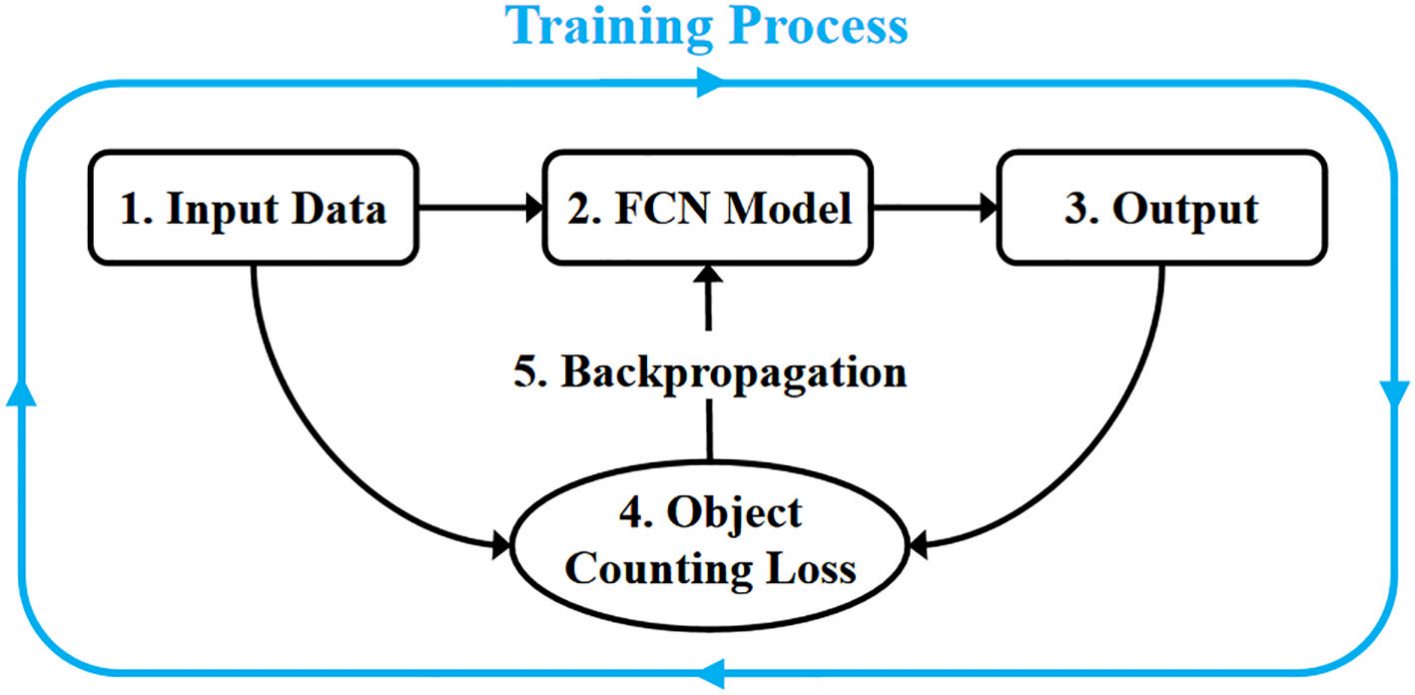
Training process of TCD-Net.

A pure fully convolutional network predicts the target’s mask during training to enable precise segmentation of target pixels, but it lacks the capability for counting and detecting targets. Zhang et al. (2024a) observed that existing object detection methods struggle to count tiny pests, as the model struggles to learn the precise location and contours of the target due to missing features, leading to poor performance. They proposed RPH-Counter, using Object Counting Loss (OC Loss) to train the fully convolutional network and incorporating a self-attention mechanism to enhance the model’s feature extraction capability, achieving precise detection of field planthoppers. Thrips are even smaller than planthoppers, presenting a greater challenge to model performance. Therefore, we further optimized the model and used OC Loss to train the fully convolutional network to enhance the detection performance for thrips. Our method uses object-level annotations similar to object detection, and the training process of the network model is shown in **Figure 9**.

The OC Loss optimizes the model’s prediction of object centers by focusing on the center points, restricting the model’s prediction range for each object according to the annotated bounding box, and continuously constraining false positives during training, as shown in Equation 3. The three sub-goals are optimized together during training, extending the original semantic segmentation capability of the fully convolutional network to include object detection and counting.


(3) $${ℒ}_{OC}\left(P,T_{L},T_{B}\right)=\underset{\text{Localization}\;\text{loss}}{\underset{︸}{{ℒ}_{L}\left(P,T_{L}\right)}}+\underset{\text{Boundary}\;\text{loss}}{\underset{︸}{{ℒ}_{B}\left(P,T_{B}\right)}}+\underset{\text{False}\;\text{positive}\;\text{loss}}{\underset{︸}{{ℒ}_{F}\left(P,T_{L}\right)}}$$

After forward propagation, the model generates a prediction matrix *P*, which has the same size as the input image. For each pixel *i*, the raw output value is denoted as $p_{i}$ . To convert this into a probability score, the *Sigmoid* activation function is applied to the model’s output. Let $P_{i}=1/\left(1+\text{exp}\left(-p_{i}\right)\right)$ be the *Sigmoid* probability of thrip at pixel *i*, the closer the value is to 1, the higher the likelihood that the position corresponds to a thrip.

Two ground-truth matrices, *T_L_* and *T_B_*, are defined, both matching the size of the input image. Matrix *T_L_* stores the center locations of pests, assigning a value of 1 to the exact center of each pest and 0 to all other pixels. This serves as a precise localization target during training. On the other hand, *T_B_* represents the object boundaries, assigning a value of 0 to pixels within the annotated bounding boxes and 1 to all other regions. This matrix is designed to guide the model in distinguishing pest boundaries from their surrounding areas. In the following sections, we will provide a comprehensive breakdown of the three sub-loss functions, each tailored to address specific aspects of the training objective.

#### 3.2.1 Localization loss

Bearman et al. (2016) proposed a point-supervised semantic segmentation loss function that only requires point-level annotations to achieve approximate object contour segmentation. We applied and integrated this loss function into the Localization loss component of the OC Loss, enabling the model to accurately localize objects. The Localization loss optimizes the model to predict a region around each object’s center, granting the model localization capabilities, as shown in Equation 4.


(4) $${ℒ}_{L}\left(P,T_{L}\right)=-\sum_{i\in {\mathbb{Z}}_{T_{L}}}\lambda_{L}\text{log}\left(P_{iT_{Li}}\right)$$

Based on the object bounding box annotations, we first compute the coordinates of each object’s center point and generate the ground truth matrix *T_L_* for the object center points. The target center point label is 1, let ${\mathbb{Z}}_{T_{L}}$ be the set of coordinates in *T_L_* where the label is 1. For these coordinates with label value of 1, let the predicted value of the corresponding position in the model prediction result *P* be $P_{iT_{Li}}$ . We aim to ensure that the model’s output value at these positions is close to 1. This optimization objective ensures that the model can accurately localize each thrips. To provide more comprehensive training, we introduce a dynamic parameter $\lambda_{L}=sum\left(T_{L}\right)$ , where the contribution to the loss increases with the number of targets in the image.

#### 3.2.2 Boundary loss

Localization loss only optimizes the model’s prediction of each object’s center region but does not provide guidance or constraints on the predicted region’s boundaries, which can lead to model “laziness”, resulting in a lack of constraint on the predicted region. Boundary loss constrains the model’s predicted range using the boundary information from the annotated bounding boxes, ensuring that the model predicts a small region around each thrips center. We pre-load a matrix *T_B_* containing the boundary information for all targets in the Dataloader. In this matrix, the value of the element corresponding to the target bounding box position is 1, and only these positions hold a value of 1. *T_B_* can indicate the boundary coordinates of each target.

Let ${\mathbb{Z}}_{T_{B}}$ be the set of coordinates in *T_B_* where the label is 1. For these boundary coordinates, let the predicted value of the corresponding position in the model prediction result *P* be $P_{iT_{Bi}}$ . We aim to ensure that the model’s output at these positions is close to 0. Boundary loss is formulated as in Equation 5.


(5) $${ℒ}_{B}\left(P,T_{B}\right)=-\sum_{i\in {\mathbb{Z}}_{T_{B}}}\lambda_{B}\text{log}\left(1-P_{iT_{Bi}}\right)$$

This optimization objective constrains the model’s predicted range, ensuring that the center of the predicted region for each object is accurate. Similarly, we introduce a dynamic parameter $\lambda_{B}=sum\left(T_{L}\right)-1$ . When the image contains more targets, the targets may be closer to each other. Therefore, we assign higher weight to Boundary Loss to ensure that each target remains independently detected.

#### 3.2.3 False positive loss

Localization loss and Boundary loss contribute only to the model’s prediction of positive samples, without encouraging the model to learn the characteristics of negative samples. Therefore, we also incorporate False Positive Loss to train the model’s ability to detect negative samples. The procedure for this is as follows: during training, we identify regions that the model incorrectly predicts as positive samples and encourage the model to predict these regions as background, as described in Equation 6.


(6) $${ℒ}_{F}\left(P,T_{L}\right)=-\sum_{i\in {\mathbb{Z}}_{F}}\text{log}\left(1-P_{i0}\right)$$

The process for calculating erroneous prediction regions is as follows: First, we use a connected component labeling algorithm to assign unique labels to each independent region in the predicted result *P*. Then, we element-wise multiply *P* with the ground truth center point matrix *T_L_* to obtain the prediction regions that contain ground truth target centers. Finally, the remaining regions are identified as erroneous predictions. Let ${\mathbb{Z}}_{F}$ be the set of coordinates in *P* corresponding to these erroneous regions. We aim for the model’s output at these positions to be close to 0.

### 3.3 Thrip counting and detection

#### 3.3.1 Thrip counting

The counting of thrips is achieved by calculating the number of independent regions in the model’s prediction *P*. This is done using a connected component labeling algorithm (He et al., 2017), specifically implemented using the *label* method from the Scipy library.

#### 3.3.2 Thrip detection

Thrip localization and detection results are obtained by calculating the centroid coordinates of each independent region. First, we extract the set of non-zero labels from the labeled matrix, excluding the background. Then, we construct a 2D coordinate matrix with the same dimensions as the input, where each pixel’s row and column indices are recorded. Both the labeled matrix and coordinate matrix are flattened into 1D arrays for vectorized computation. Histogram statistics are used to count the number of pixels for each label, and weighted accumulation is performed on the row and column coordinates to obtain the total vertical and horizontal coordinates for the pixels in each connected region. Finally, the centroid coordinates are computed for each label using the centroid calculation formula.

## 4 Experimental results

### 4.1 Implementation details

The hardware used for model training and inference consists of an Intel Core I9 12900K CPU with 64GB of memory and an NVIDIA RTX 4090 GPU. The operating system is Ubuntu 22.04.1 LTS, with CUDA version 12.1. The model is built on Python 3.9 and PyTorch 2.1.2.

#### 4.1.1 Model details

In PartialNeXt, the ratio between feature maps processed by PConv for feature extraction and those directly bypassed is 1:3, with a kernel size of 7. The downsampling rates for C2–C5 feature maps are 4×, 8×, 16×, and 32×, with channel counts of 80, 160, 320, and 640, respectively. When calculating the mixed attention for C2-C5, the local size for each layer is 32, 16, 8, and 4, respectively. The kernel size for the Conv1D in channel attention is 3, while the kernel size for Conv2D in spatial attention is also 3. All feature maps are adjusted to 256 channels in the FPN, outputting four multi-scale features with 256 channels. Finally, four 1×1 convolutions reduce the channel count of the four multi-scale features to 1, which is then resampled back to the input size and merged, with the *Sigmoid* activation function applied, resulting in the final prediction.

#### 4.1.2 Details of the methods used for comparison

We compare TCD-Net with existing methods, including one-stage detectors: YOLOv8 and YOLOv11 (Sharma et al., 2024). Two-stage detectors include Faster R-CNN (Ren et al., 2015), Cascade R-CNN (Cai and Vasconcelos, 2018) and Dynamic R-CNN (Zhang et al., 2020a). DETR-based detectors include Deformable DETR (Zhu et al., 2020) and DDQ-DETR (Zhang et al., 2023). We also compare with the recently proposed RPH-Counter (Zhang et al., 2024a). YOLO is implemented using the official open-source code, with the Large version of the model. The two-stage detectors and DETR-based detectors are implemented using the MMDetection framework, with the backbone network using ResNet50 pre-trained on ImageNet 1K. For anchor-based detectors, the anchor generation size is adapted to the target size of the rice planthopper dataset.

#### 4.1.3 Training details

During training, random flipping is used for data augmentation. The batch size is set to 1, and the Adam optimizer is used with a learning rate of 1e-5 and weight decay of 1e-4. All methods are trained for 100 epochs.

### 4.2 Evaluation metrics

#### 4.2.1 Detection accuracy

The model’s localization accuracy can be evaluated by checking whether the predicted region’s centroid lies within the ground truth bounding box. Object detection methods determine this by calculating the center point of the predicted box. The criteria for TP, FP, and FN are shown in **Table 2**.

**Table 2:** Criteria for determining TP, FP, and FN.

Flag	Description
True positive (TP)	The centroid of the predicted region lies within the ground truth bounding box
False positive (FP)	The centroid of the predicted region does not lie within any ground truth bounding box
False negative (FN)	There is no centroid of the predicted region within the ground truth bounding box

The model’s detection accuracy is evaluated using Precision, Recall, and F1 score, as shown in Equations 7-9. Our method uses a confidence threshold of 0.5, while the confidence threshold for object detection methods is determined by finding the value corresponding to the highest F1 score on the Precision-Recall curve.


(7) $$F1=\frac{2TP}{2TP+FP+FN}$$

(8) $$Precision=\frac{TP}{TP+FP}$$

(9) $$Recall=\frac{TP}{TP+FN}$$

#### 4.2.2 Counting error

The algorithm’s stability is evaluated using the Mean Absolute Error (MAE) and Root Mean Squared Error (RMSE). Let $C_{i}^{GT}$ and $C_{i}^{Pred}$ represent the ground truth and predicted number of targets in the *i-th* image, respectively. *N* be the number of images. The calculations are shown in Equations 10 and 11.


(10) $$MAE=\frac{1}{N}\sum_{i=1}^{N}\left|C_{i}^{GT}-C_{i}^{Pred}\right|$$

(11) $$RMSE=\sqrt{\frac{1}{N}\sum_{i=1}^{N}{\left|C_{i}^{GT}-C_{i}^{Pred}\right|}^{2}}$$

R-squared (R²) evaluates the similarity between the algorithm’s counting results and the actual results, as shown in Equation 12, $\bar{C^{GT}}=Avg(\sum C_{i}^{GT})$ . The R^2^ value ranges from 0 to 1, with higher values indicating that the algorithm more accurately reflects the pest situation.


(12) $$R^{2}=\frac{\sum {\left(C_{i}^{Pred}-\bar{C^{GT}}\right)}^{2}}{\sum {\left(C_{i}^{GT}-\bar{C^{GT}}\right)}^{2}}$$

### 4.3 Training results

**Figure 10:**
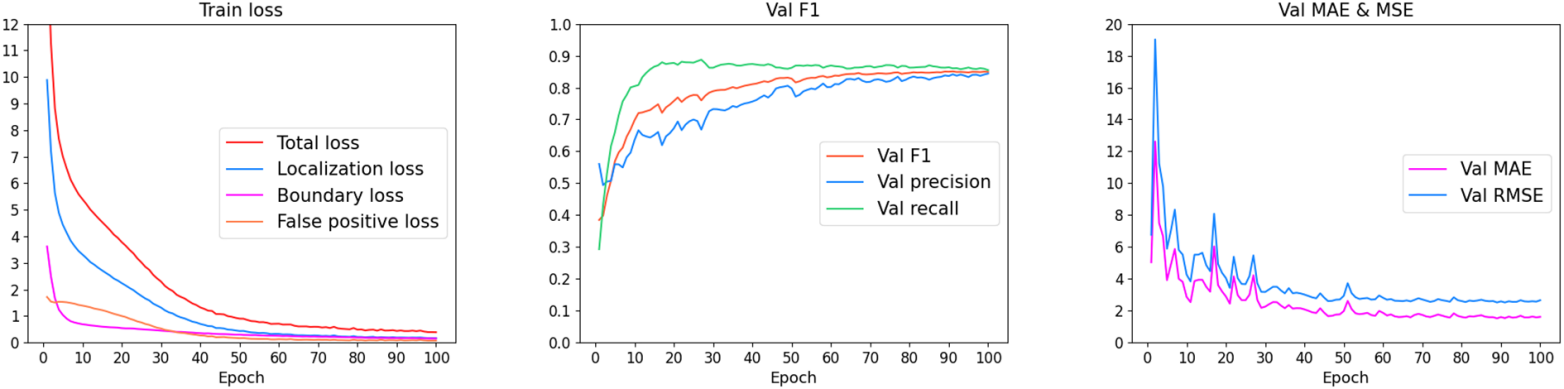
Visualization results of the training process.

We visualized the reduction in loss during training, as well as the changes in counting error and accuracy on the validation set, as shown in **Figure 10**. First, the model’s training loss steadily decreased, with all sub-loss functions being well optimized. Meanwhile, in each evaluation cycle, the counting error on the validation set generally showed a decreasing trend, while the F1 score showed an increasing trend. This indicates that, after training, the model successfully achieved the objective of detecting and counting thrips in the images.

**Figure 11:**
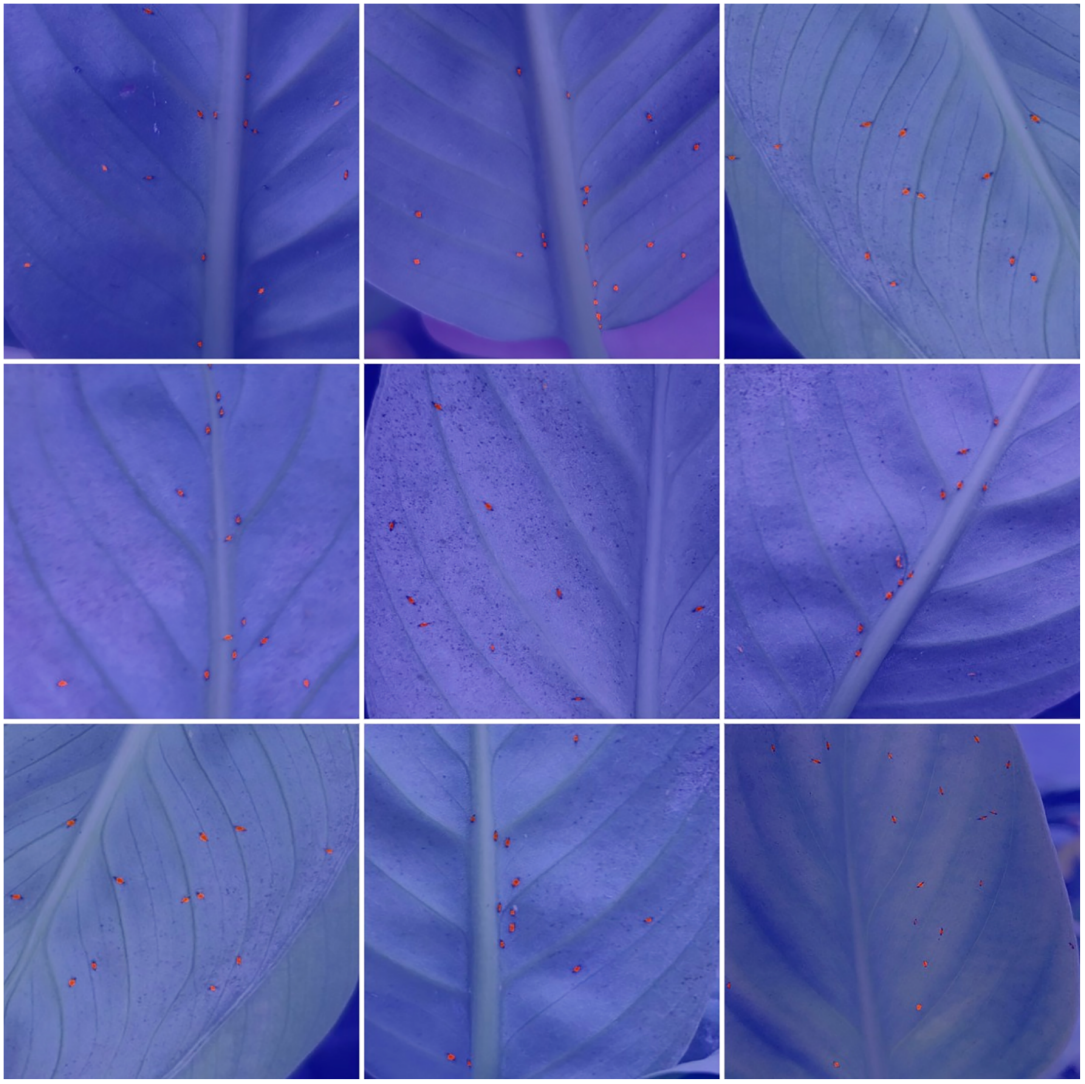
Visualization of model output.

We further visualized the model’s prediction, presented in the form of heatmaps, as shown in **Figure 11**. After sufficient training, the model demonstrated the ability to detect thrips while being insensitive to the background. For each thrip, the model predicts a small spot area, and the predicted range is confined within the thrip’s body size. Subsequently, the number of independent regions can be calculated using a connected component labeling algorithm, and by calculating the centroid of each region, precise detection and counting of thrips can be achieved.

### 4.4 Quantitative analysis

We compared TCD-Net with some existing methods widely used for pest counting. First, we compared the detection performance of these models, and the results are shown in **Table 3**. TCD-Net significantly outperforms the one-stage detectors, with both higher Precision and Recall. YOLOv8l and YOLOv11l show relatively weaker performance, with lower F1 scores and Recall rates compared to other methods, likely due to their inadequate small object detection performance. The two-stage detectors performed relatively better in the thrips detection task. Compared to the one-stage detectors, the two-stage detectors showed a significant improvement in Recall. However, their drawback lies in lower Precision, which leads to more false positives, resulting in suboptimal F1 scores. Deformable DETR achieved higher detection performance, with a primary advantage in Precision. However, due to the global attention mechanism of the Transformer, small object sparse features are prone to being overwhelmed by the background when calculated on high-dimensional feature maps. Additionally, the one-to-one matching (O2O) of predicted boxes in DETR results in far fewer positive samples than the one-to-many matching (O2M) in traditional detectors, which may reduce performance in small object detection tasks (Shihua et al., 2025). Therefore, current DETR-based detectors still face significant limitations in detecting extremely small objects, with lower Recall in thrips detection leading to many missed detections. TCD-Net demonstrated the best overall performance on both the validation and test sets, with an F1 score significantly higher than other methods. Moreover, it achieved a good balance between Precision and Recall.

**Table 3:** Comparison of detection accuracy with existing methods.

Method	Val			Test		
	F1	Precision	Recall	F1	Precision	Recall
TCD-Net	86.20%	85.30%	87.12%	85.67%	85.18%	86.17%
RPH-Counter	83.14%	83.35%	82.93%	82.98%	82.67%	83.29%
Faster R-CNN	80.68%	78.41%	83.10%	80.91%	79.31%	82.58%
Cascade R-CNN	81.05%	78.66%	83.59%	81.11%	79.80%	82.46%
Dynamic R-CNN	81.29%	81.69%	80.88%	81.23%	82.63%	79.89%
Deformable DETR	82.67%	86.22%	79.40%	82.43%	86.09%	79.07%
DDQ-DETR	82.97%	85.02%	81.02%	82.28%	84.13%	80.52%
YOLOv8l	77.52%	76.35%	78.73%	77.04%	75.90%	78.22%
YOLOv11l	76.89%	75.83%	77.98%	76.48%	75.56%	77.43%

We further compared the counting accuracy of these methods, and the results are shown in **Table 4**. TCD-Net once again demonstrates its advantage, with the lowest MAE and RMSE and the highest R^2^ value, indicating that its counting results are the closest to the actual values, with the best stability. Considering the detection accuracy results, models with higher detection performance also show higher counting accuracy, reflecting a more accurate assessment of pest conditions.

**Table 4:** Comparison of counting accuracy with existing methods.

Method	Val			Test		
	MAE	RMSE	R^2^	MAE	RMSE	R^2^
TCD-Net	1.43	2.43	76.80%	1.49	2.48	75.50%
RPH-Counter	1.69	2.66	65.62%	1.73	2.75	65.41%
Faster R-CNN	2.12	3.14	62.57%	2.13	3.17	62.23%
Cascade R-CNN	2.09	3.08	64.59%	2.09	3.06	62.85%
Dynamic R-CNN	1.96	2.99	63.54%	2.02	3.10	60.51%
Deformable DETR	1.99	2.87	65.24%	2.01	2.99	64.78%
DDQ-DETR	1.88	2.85	66.13%	2.03	3.01	64.63%
YOLOv8l	2.14	3.22	60.67%	2.15	3.26	59.73%
YOLOv11l	2.19	3.29	59.92%	2.21	3.34	58.61%

Finally, we compared the computational complexity of these methods. The comparison was based on four aspects: model parameter count, computational load, training speed, and inference speed, with the results shown in **Table 5**. When comparing with the one-stage detection models, YOLOv8 and YOLOv11, TCD-Net has lower theoretical parameter count and computational load. It also has slightly faster training and inference speeds than YOLO, while achieving significantly better detection performance. For more complex models, such as Deformable DETR and RPH-Counter, the detection performance of these models is slightly lower than that of TCD-Net, but their computational complexity is significantly higher, especially Deformable DETR, which fails to meet real-time inference speeds. In comparison with RPH-Counter, TCD-Net’s computational load is less than half, and its inference speed is approximately 1.5 times faster. In summary, TCD-Net not only achieves higher detection and counting accuracy but also maintains a relatively low computational load, with its inference speed surpassing the real-time detection requirement.

**Table 5:** Comparison of model complexity with existing methods.

Method	Params (M)	FLOPs (G)	Training speed (it/s)	Inference FPS on GPU	Inference FPS on CPU
TCD-Net	21.13	114.36	20.76	91.66	1.67
RPH-Counter	36.37	247.58	14.06	62.66	0.94
Faster R-CNN	41.35	322.42	13.21	38.76	0.16
Cascade R-CNN	69.16	350.22	11.52	34.36	0.16
Dynamic R-CNN	41.75	323.60	12.92	38.46	0.17
Deformable DETR	41.21	319.21	3.45	14.86	0.24
DDQ-DETR	48.31	437.31	3.09	12.95	0.15
YOLOv8l	43.63	275.32	15.62	52.44	0.53
YOLOv11l	25.31	147.46	17.04	74.29	0.75

### 4.5 Visualization

**Figure 12:**
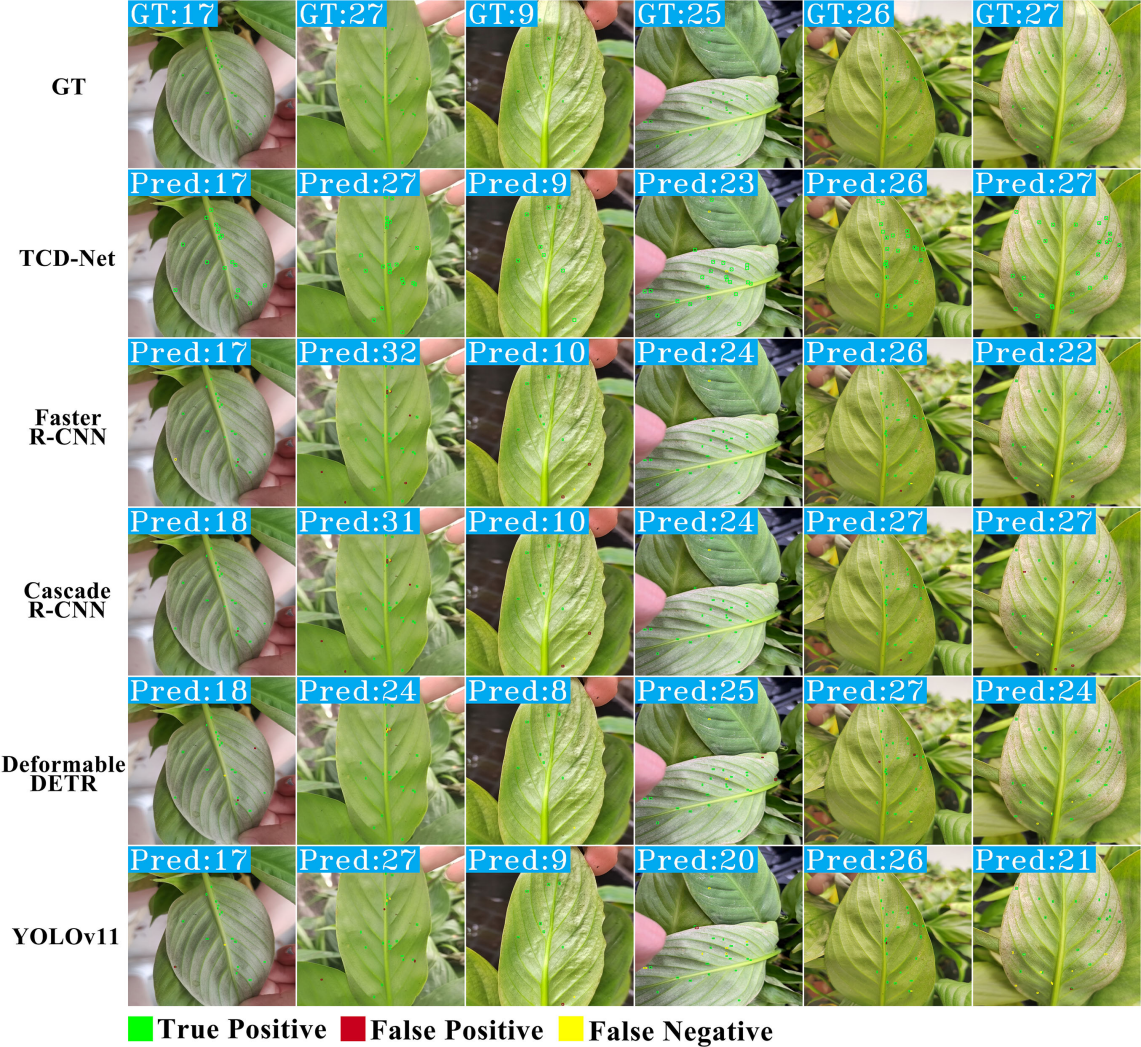
Visual comparison of prediction results with existing methods.

We visualized the detection and counting results of each method for a more intuitive comparison, as shown in **Figure 12**. Upon observing the detection results of Faster R-CNN and Cascade R-CNN, it is evident that they suffer from insufficient detection precision, with many FPs present. YOLOv11l’s detection results also include noticeable FN and FP, leading to higher counting discrepancies in some cases. The detection results of Deformable DETR contain fewer FN and FP compared to one-stage and two-stage detectors, but due to its lower recall rate, the counting results are fewer than the actual number of targets. Compared to existing methods, TCD-Net has fewer FN and FP, and its counting results are closer to the actual numbers. However, in some cases, the target detection method may exhibit significant missed detections and false detections, as shown in **Supplementary Figure S1**. This is primarily due to the small proportion of thrips’ features, making accurate identification difficult. The visualized results align with the quantitative analysis, further confirming the comprehensive advantage of TCD-Net in the thrips detection and counting task.

**Figure 13:**
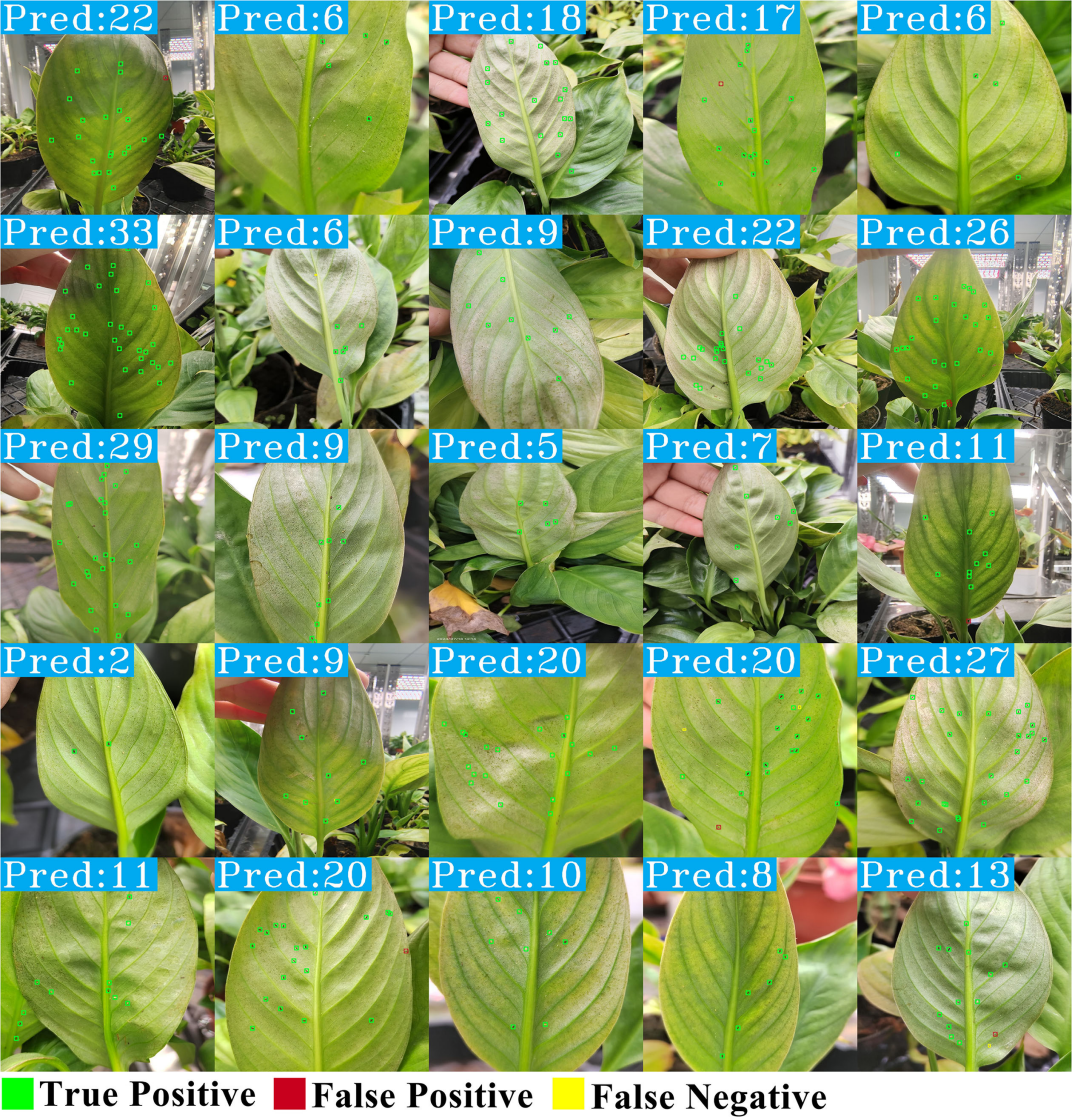
Visualization of the prediction results of TCD-Net.

Finally, as shown in **Figure 13**, we present a set of detection and counting results from TCD-Net. TCD-Net demonstrates high stability, with only a small number of FN and FP in the detection results, providing strong algorithmic support for the intelligent monitoring and management of thrips.

### 4.6 Comparative analysis

#### 4.6.1 Comparison of backbone

First, we compared PartialNeXt with several existing backbone networks, without using any attention mechanisms in the network. The features from four levels of the backbone network were input into the vanilla FPN for feature fusion. The performance comparison results are shown in **Table 6**. When using PartialNeXt, the model outperforms several existing backbone networks in terms of F1 score, RMSE, and R^2^ on both the validation and test sets. Compared to ConvNeXtV2-Nano, after applying PConv, the model’s performance significantly improves, demonstrating that using PConv is a better choice than DWConv. When compared to classic backbone networks such as ResNet-50 (He et al., 2016), Swin Transformer-Tiny (Liu et al., 2021b), and FasterNet-S (Chen et al., 2023), PartialNeXt, through lightweight design and PConv optimization, is able to extract richer features. At the same time, PartialNeXt maintains a high degree of lightweight efficiency, as shown in **Supplementary Table S1**. Compared to the larger backbone network Swin Transformer-Tiny, PartialNeXt achieves higher performance and a 4.5× faster inference speed. In comparison with ConvNeXtV2-Nano, PartialNeXt delivers significantly higher performance with minimal efficiency loss.

**Table 6:** Performance comparison of backbone.

Backbone	Val			Test		
	F1	RMSE	R^2^	F1	RMSE	R^2^
ResNet-50	82.66%	2.77	65.14%	81.91%	3.01	63.79%
Swin Transformer-Tiny	82.99%	2.75	65.21%	82.57%	2.99	64.21%
ConvNeXt-Tiny	82.84%	2.78	65.09%	82.20%	2.96	63.88%
ConvNeXtV2-Nano	82.91%	2.77	64.99%	82.21%	2.94	62.82%
FasterNet-S	82.58%	2.88	66.42%	81.80%	3.07	59.99%
PartialNeXt	83.69%	2.79	71.25%	83.56%	2.87	65.87%

#### 4.6.2 Comparison of attention mechanism

Next, we fixed the backbone network as PartialNeXt and used the vanilla FPN for feature fusion. We compared the performance and efficiency of different attention mechanisms. The performance comparison results are shown in **Table 7**. When using our proposed HA to process the multi-level feature maps of the backbone network, model performance improves, especially in R^2^, which shows a notable enhancement. This indicates that, after using HA, the model’s output becomes more stable. Meanwhile, as shown in **Supplementary Table S2**, the computational cost of HA is lower than that of CBAM, and the inference speed is only slightly lower than MLCA, achieving a good balance between model performance and efficiency.

**Table 7:** Performance comparison of attention mechanism.

Attention	Val			Test		
	F1	RMSE	R^2^	F1	RMSE	R^2^
-	83.69%	2.79	71.25%	83.56%	2.87	65.87%
CBAM	85.12%	2.67	72.44%	84.20%	2.68	67.17%
MLCA	84.81%	2.64	71.46%	84.37%	2.58	68.99%
HA	85.54%	2.46	75.34%	84.94%	2.61	69.04%

#### 4.6.3 Comparison of FPN

Finally, we compared the performance and efficiency of different FPNs. With the backbone network fixed as PartialNeXt and no attention mechanism, the performance comparison results are shown in **Table 8**. Using our proposed AFM-FPN further enhanced the model’s feature fusion mechanism, improving the model’s detection and counting performance for thrips. At the same time, as shown in **Supplementary Table S3**, the model with AFM-FPN has lower parameters and computational cost, balancing model performance and efficiency effectively.

**Table 8:** Performance comparison of FPN.

FPN	Val			Test		
	F1	RMSE	R^2^	F1	RMSE	R^2^
Vanilla FPN	83.69%	2.79	71.25%	83.56%	2.87	65.87%
PAFPN	85.05%	2.48	72.27%	84.58%	2.62	70.01%
AFM-FPN	85.93%	2.45	75.75%	85.35%	2.55	71.03%

### 4.7 Ablation study

#### 4.7.1 Loss ablation

**Figure 14:**
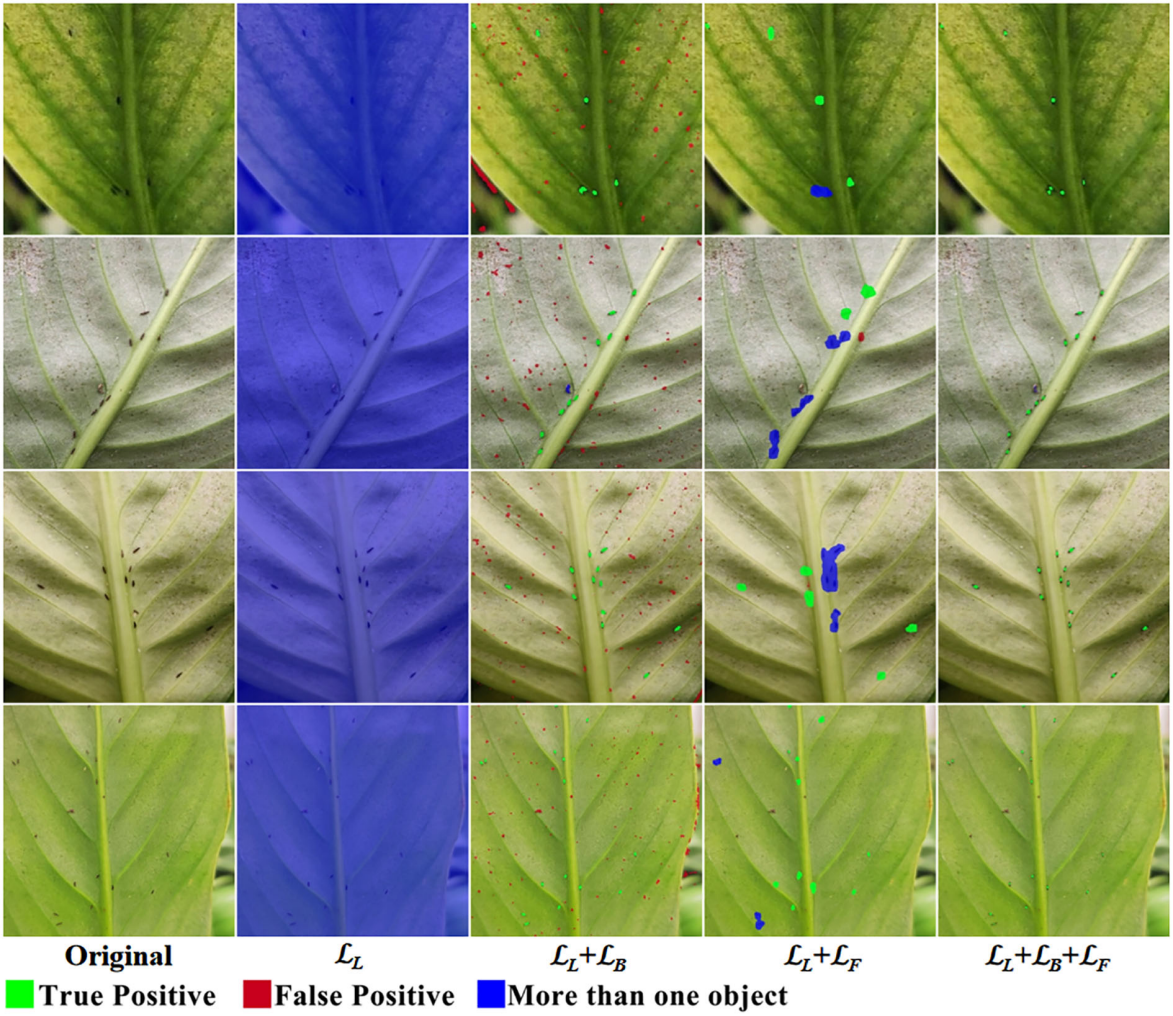
Visual comparison of loss ablation.

We conducted an ablation study on the components of the loss function, and the visualization results are shown in **Figure 14**. When only ℒ*_L_* is used, the model exhibits “laziness,” predicting the entire image as the foreground to include all thrips. When ℒ*_L_* +ℒ*_B_* is used, the lack of constraints on false positives leads to a large number of false positive predictions. When ℒ*_L_* +ℒ*_F_* is used, the model predicts a larger spot for each thrips, but due to the absence of constraints on prediction boundaries, the model is unable to separate thrips that are close together. When the complete loss function is used, the model predicts a smaller spot for each thrips, with individuals well separated, and false positives are constrained, achieving precise detection and counting of thrips.

#### 4.7.2 Network module ablation

We conducted ablation experiments on the three key improvements we proposed to validate their effectiveness, and the model performance comparison results are shown in **Table 9**. Each of the three proposed improvement modules effectively enhances the model’s performance, and when combined, they exhibit significant synergistic effects. First, when used individually, each of these modules improves the evaluation metrics, confirming the independent effectiveness of each module. Then, combining two modules further enhances performance, with PConv+AFM-FPN performing the best, showing an 8.21% improvement in R^2^ on the test set. Finally, when all three improvements are combined, the model achieves optimal performance, with the test set F1 reaching 85.67%, RMSE reduced by 15.6%, and R^2^ increased by 12.68%. These results significantly outperform the baseline and any combination of submodules, demonstrating the rationality and necessity of the multi-module collaborative design.

**Table 9:** Ablation study on model performance.

PConv	HA	AFM-FPN	Val			Test		
			F1	RMSE	R2	F1	RMSE	R2
			82.91%	2.77	64.99%	82.21%	2.94	62.82%
✓			83.69%	2.79	71.25%	83.56%	2.87	65.87%
	✓		83.20%	2.73	66.97%	82.66%	2.83	64.55%
		✓	83.68%	2.66	68.49%	83.07%	2.90	65.88%
✓	✓		85.54%	2.46	75.34%	84.94%	2.61	69.04%
✓		✓	85.93%	2.45	75.75%	85.35%	2.55	71.03%
	✓	✓	84.39%	2.55	72.70%	83.60%	2.72	67.36%
✓	✓	✓	86.20%	2.43	76.80%	85.67%	2.48	75.50%

As shown in **Table 10**, we further investigated the impact of these improvements on the model’s computational efficiency. First, PConv, due to the use of partial vanilla convolution, results in a noticeable increase in parameters and computational load (+5M Params, +25.96G FLOPs), but has minimal impact on training speed. HA, with almost no increase in parameters and computation, slightly reduces the inference speed, indicating that the computational overhead of its attention mechanism is manageable. The multi-scale fusion structure introduced by AFM-FPN also only slightly increases the computational burden (+0.2M Params, +4.49G FLOPs), while maintaining high training and inference efficiency. When combining the modules, the inference speed drops to 91.66 it/s but still exceeds the real-time requirements. Overall, the modules achieve a good balance between computational cost and performance improvement.

**Table 10:** Ablation study on model efficiency.

PConv	HA	AFM-FPN	Params (M)	FLOPs (G)	Training speed (it/s)	Inference speed (it/s)
			15.93	83.89	21.69	113.02
✓			20.93	109.85	21.32	106.07
	✓		15.93	83.91	21.35	107.08
		✓	16.13	88.38	21.44	108.11
✓	✓		20.93	109.87	21.13	100.56
✓		✓	21.13	114.34	20.99	96.31
	✓	✓	16.13	88.40	21.18	97.54
✓	✓	✓	21.13	114.36	20.76	91.66

## 5 Discussion

We identified the performance shortcomings of existing methods in thrips detection and made key improvements to address these issues. The main advantages of TCD-Net include: 1) State-of-the-art optimization: TCD-Net follows the latest neural network optimization approaches, improving the model’s performance through enhancements in feature extraction, attention mechanisms, and multi-scale feature fusion. 2) Model efficiency: While optimizing the model, we ensure its efficiency by using methods with low parameter and computational requirements, rather than merely stacking modules, achieving a balance between performance and efficiency. 3) Specialized loss function: We use a loss function tailored for small object pest detection, avoiding the issue in traditional object detection methods where it is difficult to predict and match precise small target bounding boxes, ensuring the model’s baseline performance.

However, this work still faces some limitations. First, regarding the dataset, we have collected a thrips dataset with over 47K+ annotations in a greenhouse, and the public release of this dataset can contribute to the field of extremely small pest detection. Although TCD-Net has shown good performance in our environment, greenhouse and field conditions are nearly infinitely complex, and the diversity and scale of the dataset still require further development. While data collection and annotation took considerable time and incurred high labor costs, it remains crucial to gather richer datasets in future work. New data augmentation techniques can be explored, such as using generative models like GANs and diffusion models to synthesize new data, which can be combined with the original dataset, reducing annotation costs and increasing data richness (Lu et al., 2022; Zhang et al., 2024b). Furthermore, unsupervised and weakly supervised methods can be explored for model training to reduce the need for large annotated datasets and enhance model generalization (Bollis et al., 2022; Han et al., 2025).

Regarding method optimization, further development of the model’s attention and feature fusion mechanisms is an ongoing direction that requires continued exploration. At the same time, model efficiency must be considered to ensure feasibility in practical deployment. The loss function also needs further development. While it has been successful for small pest counting, its current support for large-scale, multi-class tasks is limited. Future work could focus on optimizing the localization loss part of the loss function to enhance its multi-class support capabilities. Another potential avenue is the development of hybrid or multi-branch networks to improve support for large-scale pest detection. For example, using a hybrid machine learning and deep learning structure could enhance model performance, or employing a combined density estimation and object detection network could simultaneously improve pest detection and counting accuracy (Gao et al., 2024; Han et al., 2024).

## 6 Conclusions

This paper presents an efficient model for thrips counting and detection, capable of performing real-time, accurate counting and detection of thrips on the leaves of *Spathiphyllum floribundum ‘Clevelandii’* in greenhouses. TCD-Net is a unique fully convolutional network structure, which utilizes our designed efficient PartialNeXt as the backbone network, combined with lightweight Hybrid Attention and AFM-FPN to extract and fuse rich thrips features. By predicting a small region for each thrips, TCD-Net achieves precise counting and detection. Experiments were conducted on a dataset containing over 47K thrips annotations, and the results demonstrate that TCD-Net provides highly accurate counting and detection performance, while maintaining low model complexity and an inference speed that far exceeds real-time detection. On the test set, TCD-Net achieved an F1 score of 85.67% and a counting result correlation of 75.50%, outperforming existing methods in both counting and detection accuracy. Additionally, the model size (21.13M parameters) and theoretical computational load (114.36 GFLOPs) are less than half that of two-stage object detection methods, while the inference speed (91.66 it/s) is more than twice as fast as that of two-stage object detection methods. In summary, TCD-Net achieves higher thrips counting and detection accuracy with lower computational complexity, demonstrating its potential for detecting extremely small pests. Future optimization directions include further improving model training and inference speeds, integrating with patrol robots for intelligent pest monitoring in greenhouses, and expanding its application to other types of pests, contributing to intelligent pest reporting systems.

## Data Availability

The original contributions presented in the study are included in the article/**Supplementary Material**. Further inquiries can be directed to the corresponding author/s.
