# Supplementary material for: Fast real-time detection and counting of thrips in greenhouses with multi-level feature attention and fusion
Source: Front Plant Sci. 2025 Aug 21;16:1663813. doi: 10.3389/fpls.2025.1663813 (PMC12408579; doi:10.3389/fpls.2025.1663813)
Supplement: Supplementary file 1 [file DataSheet1.docx]

**Supplementary materials**

**Figure S1. Some cases of excessive missed/false detections when using traditional object detectors**


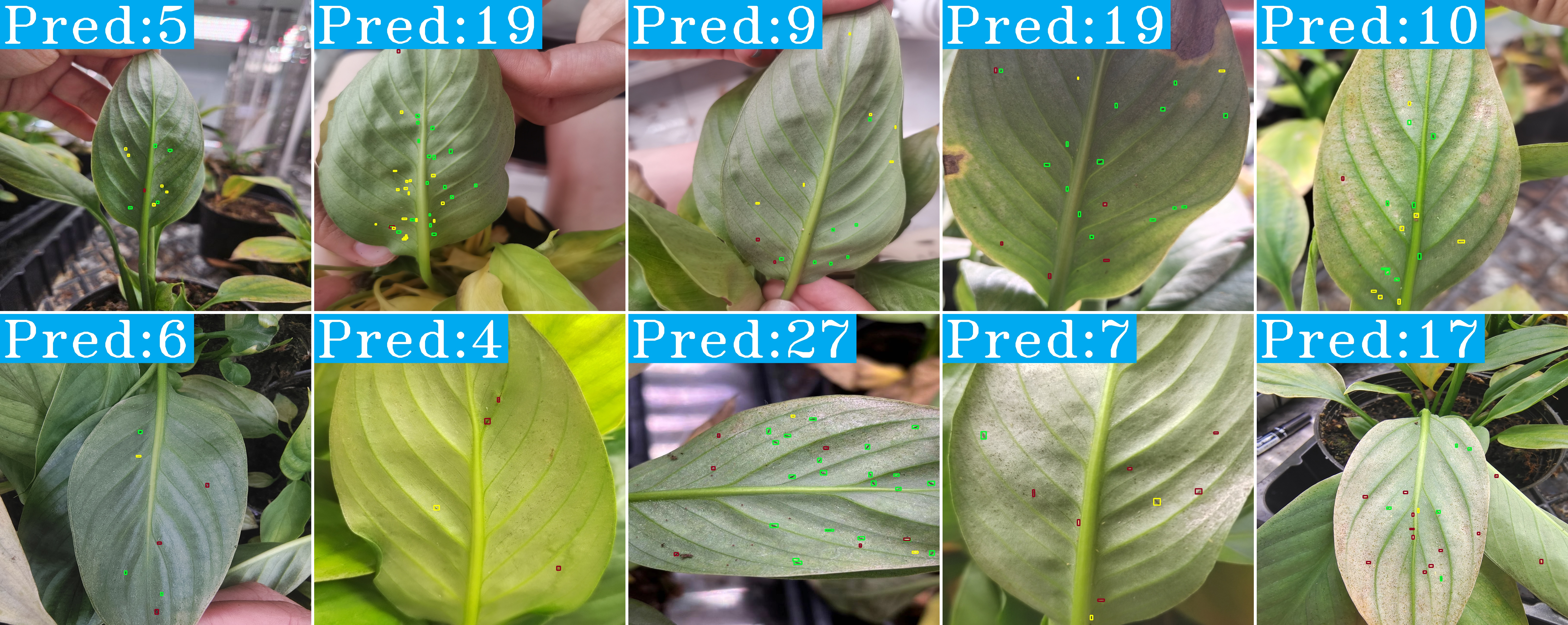


| Table S1. Efficiency comparison of backbone | | | | |
| --- | --- | --- | --- | --- |
| Backbone | Params (M) | FLOPs (G) | Training speed (it/s) | Inference speed (it/s) |
| ResNet-50 | 24.7 | 153.87 | 22.49 | 105.36 |
| Swin Transformer-Tiny | 87.4 | 339.24 | 5.61 | 23.33 |
| ConvNeXt-Tiny | 28.96 | 150.34 | 11.11 | 73.77 |
| ConvNeXtV2-Nano | 15.93 | 83.89 | 21.69 | 113.02 |
| Fasternet_S | 29.04 | 155.37 | 25.01 | 119.28 |
| PartialNeXt | 20.93 | 109.85 | 21.32 | 106.07 |

| Table S2. Efficiency comparison of attention mechanism | | | | |
| --- | --- | --- | --- | --- |
| Attention | Params (M) | FLOPs (G) | Training speed (it/s) | Inference speed (it/s) |
| - | 20.93 | 109.85 | 21.32 | 106.07 |
| CBAM | 21.07 | 109.90 | 20.31 | 85.31 |
| MLCA | 20.93 | 109.87 | 21.01 | 102.78 |
| HA | 20.93 | 109.87 | 21.13 | 100.56 |

| Table S3. Efficiency comparison of FPN | | | | |
| --- | --- | --- | --- | --- |
| Attention | Params (M) | FLOPs (G) | Training speed (it/s) | Inference speed (it/s) |
| Vanilla FPN | 20.93 | 109.85 | 21.32 | 106.07 |
| PAFPN | 22.70 | 129.69 | 20.97 | 96.35 |
| AFM-FPN | 21.13 | 114.34 | 20.99 | 96.31 |
